# Safety and Efficacy of Laparoscopic Versus Open Gastrectomy in Patients With Advanced Gastric Cancer Following Neoadjuvant Chemotherapy: A Meta-Analysis

**DOI:** 10.3389/fonc.2021.704244

**Published:** 2021-08-06

**Authors:** Xu-Liang Liao, Xian-Wen Liang, Hua-Yang Pang, Kun Yang, Xin-Zu Chen, Xiao-Long Chen, Kai Liu, Lin-Yong Zhao, Wei-Han Zhang, Jian-Kun Hu

**Affiliations:** Department of Gastrointestinal Surgery and Laboratory of Gastric Cancer, State Key Laboratory of Biotherapy, West China Hospital, Sichuan University, and Collaborative Innovation Center for Biotherapy, Chengdu, China

**Keywords:** laparoscopic gastrectomy, open gastrectomy, neoadjuvant chemotherapy, advanced gastric cancer, safety and efficacy

## Abstract

**Background:**

Given the expanding clinical applications of laparoscopic surgery and neoadjuvant chemotherapy in advanced gastric cancer treatment, there is an emerging need to summarize the few evidences that evaluated the safety and efficacy of laparoscopic versus open gastrectomy in patients with advanced gastric cancer (AGC) following neoadjuvant chemotherapy (NAC).

**Methods:**

From January 1 to 2, 2021, we searched Ovid Embase, PubMed, Cochrane central register Trials (Ovid), and web of science to find relevant studies published in English, and two authors independently performed literature screening, quality assessment of the included studies, data extraction, and data analysis. This study was registered with PROSPERO (CRD42021228845).

**Results:**

The initial search retrieved 1567 articles, and 6 studies were finally included in the meta-analysis review, which comprised 2 randomized control trials and 4 observational studies involving 288 laparoscopic gastrectomy (LG) and 416 open gastrectomy (OG) AGC patients treated with NAC. For intraoperative conditions, R0 resection rate, blood transfusion, intraoperative blood loss, number of lymph nodes dissected, proximal margin, and distal margin were comparable between LG group and open OG group. For postoperative short-term clinical outcomes, LG has significantly less postoperative complications (OR = 0.65, 95%CI: 0.42–1.00, p = 0.05) and shorter postoperative time to first aerofluxus (WMD = -0.57d, 95%CI: -0.89–0.25, p = 0.0004) than OG, and anastomotic leakage, pulmonary infection, pleural effusion, surgical site infection, thrombosis, intestinal obstruction, peritoneal effusion or abscess formation, postoperative time to first defecation, postoperative time to first liquid diet, and postoperative length of stay were comparable between the two groups. For postoperative survival outcomes, there were no significant differences in disease-free survival (DFS) and overall survival (OS) between the two groups.

**Conclusion:**

The available evidences indicated that LG is an effective and feasible technology for the treatment of AGC patients treated with NAC, and LG patients have much less postoperative complications and faster bowel function recovery than OG patients.

**Systematic Review Registration:**

PROSPERO database (identifier, CRD42021228845).

## Introduction

Gastric cancer is still one of the most common type of malignancy worldwide, with its morbidity and tumor-related mortality ranking fifth and fourth, respectively, among all kinds of cancers. Notably, gastric cancer is responsible for about 770,000 deaths per year (1). Advanced gastric cancer (AGC) comprises a large proportion of all gastric cancer patients, and surgeons struggle with how to prolong overall survival (OS) and improve disease-free survival (DFS). Current therapeutic strategies for AGC comprise neoadjuvant chemotherapy (NAC) and radical surgical resection, which have to fulfill negative surgical margin and harvest sufficient number of lymph nodes (more than 16) (2–5). One study reported that operative treatment is the key step for treating progressive gastric carcinoma (6), and a positive surgical margin will significantly shorten patient survival time. Over the years, gastrointestinal surgeons have gradually applied NAC for local treatment of AGC ever since it was recommended for cancer treatment. The roles of NAC include downstage of tumor stage, elimination of micrometastases, and a better tolerance, which are able to increase the probability of radical surgery and eventually extend the survival time of cancer patients (7).

According to the available literature, laparoscopic distal gastrectomy was first implemented in 1994 in Japan, and laparoscopic-assisted billroth I gastrectomy was used for the treatment of early gastric cancer patients (8). Since then, we have witnessed the change of radical gastrectomy from traditional open surgery to laparoscopic-assisted surgery. Several randomized control trials (RCT) and meta-analysis studies have shown that laparoscopic gastrectomy (LG) has essentially the same efficacy compared to conventional open gastrectomy (OG) in treating AGC (9–16), including DFS and OS. However, the laparoscopic approach has obvious advantages over conventional laparotomy such as less trauma and faster recovery. These advantages in safety and effectiveness have led to widespread use of LG in patients with AGC.

However, the safety and efficacy of LG in patients with AGC following NAC has not yet been elucidated. The proinflammatory response induced by chemotherapy produces proinflammatory cytokines, profibrotic response, and cytotoxic reaction, thereby leading to the loss of structural integrity in the tissue and organ function, which presents a new challenge in laparoscopic surgery (17–26). Moreover, it is not clear whether smaller trauma in AGC patients who receive NAC is equivalent to better surgical effectiveness and postoperative safety, and survival benefit is still a pivotal issue in the clinic. Strikingly, although a review of literature provides direct evidence of the issues listed above (27–32), including two RCTs, one prospective study, and four retrospective studies, all the studies have reported inconsistent findings. Therefore, further meta-analysis is urgently required to test the safety and efficacy of using laparoscopic surgery as an alternative to open surgery for AGC patients who have completed NAC.

In this study, data was collected from original studies that consisted of basic characteristics, intraoperative conditions, postoperative short-term clinical outcomes, and postoperative survival outcomes. A meta-analysis was then conducted to determine outcomes of LG versus OG in patients with AGC following NAC, with results being used to prove the non-inferiority of LG compared to OG.

## Materials and Methods

### Literature Search

A systemic search was performed by two authors (Liao XL and Liang XW) on Ovid Embase, PubMed, Cochrane central register Trials (Ovid), and web of science databases to identify relevant studies published up to January 2021 according to subject words and free words adjusted to fit various databases. The search strategy framework was expressed as follows: ((((open gastrectomy OR open surgery OR laparotomy OR abdom* operation OR transabdominal surgery) OR (“Laparotomy”[Mesh])) OR ((minimally invasive gastrectomy OR laparoscop* gastrectomy OR laparoscop* surgery OR laparoscop* operation) OR (“Laparoscopy”[Mesh]))) AND (((gastric cancer OR gastric carcinoma OR stomach cancer OR stomach neoplasm* OR stomach carcinoma OR gastric tumor OR stomach tumor)) OR (“Stomach Neoplasms”[Mesh]))) AND ((neoadjuvant chemotherapy OR new adjuvant chemotherapy OR new auxiliary chemotherapy OR preoperative adjuvant chemotherapy OR neoadjuvant chemical therapy OR new supplementary chemotherapy) OR (“Neoadjuvant Therapy”[Mesh])). The retrieval language was only confined to English, and the retrieval time was limited to the dates up to 1^st^ or 2^nd^ January 2021. To ensure inclusion of all relevant studies, references from the selected literature were retrieved by manually searching to find additional studies that were not detected in the previous literature search. This meta-analysis was conducted in accordance with PRISMA statement (33). Notably, ethical approval from ethics committees or ethics boards was not necessary because no individual patient was involved in this meta-analysis. The protocol used in this study was registered on PROSPERO database with registration number CRD42021228845.

### Literature Screening

After completing the preliminary search and removing duplicates, two authors (Liao XL and Liang XW) independently reviewed the retrieved articles according to their titles and abstracts in order to identify the potentially relevant studies for further assessment. Next, full texts of the eligible studies were downloaded, and a full-text screening was performed by two authors (Liao XL and Liang XW) based on the inclusion and exclusion criteria. All discrepancies were resolved by consensus and then checked by a third reviewer (Hu JK).

### Inclusion and Exclusion

All studies selected for the meta-analysis had to fulfill the following inclusion criteria: (1) patients with AGC diagnosed by histopathologic examination; (2) patients undergoing gastrectomy after completing NAC; and (3) studies that reported the number of LG patients and OG patients, respectively. Studies were excluded if they were conference abstracts, case reports, letters, editorials, reviews, and any type of study other than a peer-reviewed original research. In addition, a technique report from national public health institutes was excluded. Studies that reported other cancer instead of AGC, such as gastrointestinal stromal tumors and esophageal carcinoma, and studies that did not separate AGC from the above tumors were also excluded. With regard to two or more eligible studies conducted on the same population, the study involving multiple centers, providing more information, and involving more patients was included.

### Data Extraction

Firstly, we created a ‘basic characteristics’ table using basic characteristics of included studies to extract the basic information of studies that contribute data to this meta-analyses as the pre-specified outcomes. Next, two authors (Liao XL and Liang XW) extracted data separately from each included study, and all data were entered into the data and information extraction table, including intraoperative conditions, postoperative short-term clinical outcomes, and postoperative survival outcomes. In instances where sufficient information could not be obtained from publicly available data sources, the incomplete information was obtained by contacting the corresponding author of the original study.

### Quality Assessment

Two independent researchers assessed the methodological quality of two RCTs, one prospective study, and four retrospective studies, with disagreements being resolved by consultation. Jadad Composite Scale (JCS) was used to assess the methodological quality of RCTs (34). JCS contains four broad categories, which assess randomization, double-blinding, and description of withdrawals and dropouts. For each question in the categories, an affirmative response was awarded one point, while a negative response was awarded a zero point. A score of 0–2 was regarded as “low quality”, while a score of 3–5 was deemed as “high quality”. Moreover, the Newcastle Ottawa Quality Assessment Scale (NOS) was used to assess the quality of non-randomized controlled trial (35). NOS was classified into three categories containing selection, comparability, and exposure/outcome, which were then divided into eight entries. A maximum of one star was awarded for every high quality item of selection and exposure/outcome, and a maximum of two stars could be added into the items categorized under comparability. Finally, the included studies were classified into low quality (0–5 stars) and high quality (6–9 stars) based on the number of stars.

### Statistical Analysis

In this study, I^2^ and Q statistics were used to measure heterogeneity among the included studies. Results with I^2^>50% or P<0.1, taking into account the heterogeneity across studies, including inclusion and exclusion criteria heterogeneity, chemotherapy regimens, surgical technique heterogeneity, and medical conditions heterogeneity, were considered to have substantial heterogeneity, and thus, data synthesis analyses were carried out using a random-effects model to adjust for weighting of studies. Otherwise, the fixed-effects model was used. Weighted mean with 95% confidence intervals (CIs) was calculated for continuous variables, including intraoperative blood loss, number of dissected lymph nodes, proximal margin, distal margin, postoperative time to first aerofluxus, postoperative time to first defecation, postoperative time to first liquid diet, and postoperative length of stay. On the other hand, odds ratios (ORs) with 95% CIs were calculated for dichotomous outcomes, including R0 resection, blood transfusion, postoperative complications, anastomotic leakage, pulmonary infection, pleural effusion, surgical site infection, thrombosis, intestinal obstruction, and peritoneal effusion or abscess formation. Moreover, hazard ratio with 95% CIs was calculated for OS and DFS. To determine whether different studies had different effect on the overall results of the meta-analysis, sensitivity analysis was conducted by sequentially removing each single study from the meta-analysis and re-running the meta-analysis. Both the fixed and random models were performed to assess whether model selection had an important influence on results of the meta-analysis. All of the analyses were performed using RevMan 5.4.1 software (https://tech.cochrane.org/revman), Microsoft Excel 2013, and Engauge Digitizer software 11.1 (http://digitizer.sourceforge.net). In addition, all reported statistical tests were two-tailed with alpha level of 0.05.

## Results

### Literature Search and Screening

**Figure 1:**
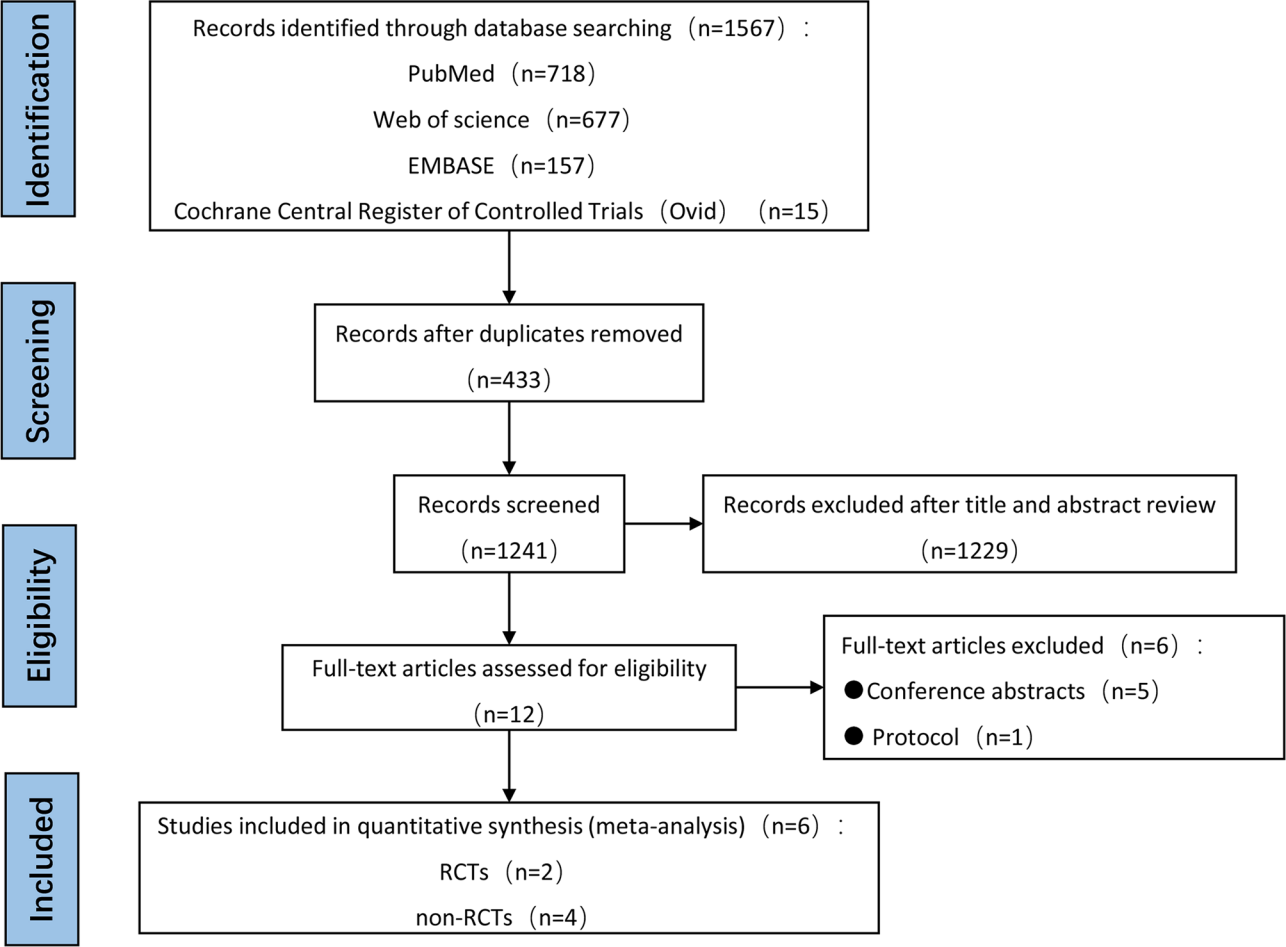
PRISMA selection flow diagram.

In total, 1567 articles were initially identified from electronic databases, of which 1241 were determined to be unique literatures after conducting automatic de-duplication. Among the 1241 articles, 1229 were excluded after title and abstract review, thereby leaving 12 articles for full-text eligibility review. Five studies were excluded because they were conference abstracts, and one protocol study was excluded due to unavailability of data. No additional studies were found after hand-searching reference lists. Finally, there were only six eligible studies that fulfilled all inclusion criteria for this meta-analysis, and thus, they were used to perform both qualitative and quantitative analyses. It is worth noting that publication bias was not statistically performed because only six studies were included. The entire systematic literature review showing the process of literature retrieval and screening was illustrated using the Preferred Reporting Items for Systematic Reviews and Meta-analyses (PRISMA) flow diagram, and is presented in **Figure 1**.

### Study Characteristics

**Table 1** shows the basic characteristics of the included studies. The six studies were published between 2016 and 2020, and they involved a total of 644 AGC patients treated with LG or OG following NAC from January 2007 to June 2018. Of the 644 patients who underwent surgery, 228 (35.4%) patients underwent LG, and 416 (64.6%) patients received OG. The NAC regimens included XELOX, FOLFOX, SOX, CAPOX, SP, TXT, TCF, DOS, ECC, ECF, EOX, FLOT, and other chemotherapy regimens. Results showed that there were no significant differences in sex, body mass index (BMI), American Society of Anaesthesiology (ASA) scores, objective response rate (ORR), clinical TNM stage, ypT3 or T4 stage, ypN2 or N3 stage, resection range, reconstruction approach, and tumor longitudinal between the LG group and OG group in the included studies (**Table 2**). In addition, AGC patients in the OG group were 1.83 years older than in the LG group. The quality of each of the six studies was evaluated using the Newcastle-Ottawa Scale (NOS) for prospective or retrospective studies and JCS for randomized controlled trials. Based on the NOS assessment, three retrospective studies received seven out of nine stars indicating high quality, and one prospective study scored eight stars also indicating high quality. Furthermore, JCS assessment showed that the two RCT studies had high quality assessment scores, three.

**Table 1:** Basic characteristics of included studies.

Reference	Country	Study interval	Study design	Sample size	Number of patients (T *vs.* C)	NACT regimen (T *vs.*. C), %	Quality assessment
Li 2019 (27)	China	2015.4–2017.11	RCT	95	47 *vs.* 48	XELOX	3 scores^*^
Li 2016 (28)	China	2012.9–2014.3	P	44	20 *vs.* 24	FOLFOX; SOX; CAPOX	8 stars^#^
Wang 2020 (29)	China	2007.1–2016.12	R	270	49 *vs.* 221	XELOX; FOLFOX; SOX; SP; TXT+XELOX; TCF; DOS; TXT+SP; Others	7 stars^#^
Fujisaki 2018 (30)	Japan	2009.11–2018.1	R	49	20 *vs.* 29	SP; SOX; tmab+SOX; tmab+CAPOX	7 stars^#^
Xi 2019 (31)	China	2013.6–2016.3	R	90	45 *vs.* 45	XELOX; SOX;	7 stars^#^
Wielen 2020 (32)	Europe	2015.1–2018.6	RCT	96	47 *vs.* 49	ECC; ECF; EOX; FOLFOX; FLOT; Others	3 scores^*^

T, Laparoscopic surgery; C, Open surgery; R, Retrospective study; P, Prospective study; RCT, Randomized controlled trial; NA, Not available; XELOX, Capecitabine and Oxalplatin; FOLFOX, Leucovorin Calcium, Fluorouracil and Oxaliplatin; SOX, Oxaliplatin, Tegafur, Gimeracil and Oteracil; CAPOX, Capecitabine and Oxalplatin; SP, Cisplatin, Tegafur, Gimeracil and Oteracil; TXT, Docetaxel; TCF, Docetaxel, Carboplatin and 5-fluorouracil; DOS, Docetaxel, Oxaliplatin, Tegafur, Gimeracil and Oteracil; ECC, Epirubicin and cyclophosphamide; ECF, Epirubicin, Cisplatin and Fluorouracil; EOX, Epirubicin, Oxaliplatin and Capecitabine; FLOT, Docetaxel, Oxaliplatin, Leucovorin and Fluorouracil.

*Jadad Composite Scale (JCS); ^#^The Newcastle Ottawa Quality Assessment Scale (NOS).

**Table 2:** The meta-analysis of clinical features of AGC patients following NAC between LG group and OG group.

Characteristics	No. of study	LG	OG	Test of heterogeneity		Model	Meta-analysis		
				I^2^ (%)	P value		OR or MD	(95%CI)	P value
Age (years)	6	228	416	0	0.90	Fixed	-1.83 years*	[-3.45, -0.21]	0.03
Sex (male)	6	228	416	0	0.53	Fixed	0.89	[0.61, 1.28]	0.52
BMI (kg/m^2^)	5	188	195	0	0.45	Fixed	0.40 kg/m2*	[-0.31, 1.11]	0.27
3 ASA scores (high risk)	4	132	147	0	0.72	Fixed	1.03	[0.54, 1.95]	0.93
ORR^#^	4	161	342	0	0.51	Fixed	1.06	[0.68, 1.67]	0.79
StageII (clinical TNM stage^&^)	5	181	367	53	0.07	Random	0.84	[0.40, 1.80]	0.66
StageIII	5	181	367	54	0.07	Random	1.08	[0.51, 2.32]	0.83
ypT3 or T4 stage	2	94	97	0	0.42	Fixed	0.96	[0.54, 1.73]	0.9
ypN2 or N3 stage	2	94	97	52	0.15	Random	1.30	[0.50, 3.41]	0.59
Proximal resection	6	228	416	0	0.63	Fixed	0.93	[0.53, 1.65]	0.81
Distal resection	6	228	416	0	0.93	Fixed	1.12	[0.75, 1.69]	0.58
Total resection	6	228	416	0	0.89	Fixed	0.93	[0.62, 1,39]	0.71
Billroth-I	3	112	117	23	0.27	Fixed	0.50	[0.24, 1.02]	0.06
Billroth-II	3	112	117	72	0.03	Random	1.46	[0.39, 5.37]	0.57
Roux-en-Y	3	112	117	66	0.09	Random	1.12	[0.36, 3.44]	0.85
Upper one-third (tumor location)	3	141	314	0	0.81	Fixed	1.10	[0.66, 1.84]	0.71
Middle one-third	3	141	314	0	0.96	Fixed	1.37	[0.82, 2.29]	0.23
Lower one-third	3	141	314	0	0.56	Fixed	0.64	[0.39, 1.04]	0.07

AGC, advanced gastric cancer; LG, laparoscopic gastrectomy; OR, Odds Ratio; MD, mean difference; BMI, body mass index; ASA, The American Society of Anesthesiologists; ORR, Objective response rate; TNM, Tumor, Node and Metastasis.

*Mean Difference (MD) was calculated; ^#^Tumor responses evaluation was performed using the Response Evaluation Criteria in Solid Tumors (RECIST) guideline (v1.0) (36); ^&^According to the Japanese Classification of Gastric Cancer (37).

### Intraoperative Conditions

**Figure 2:**
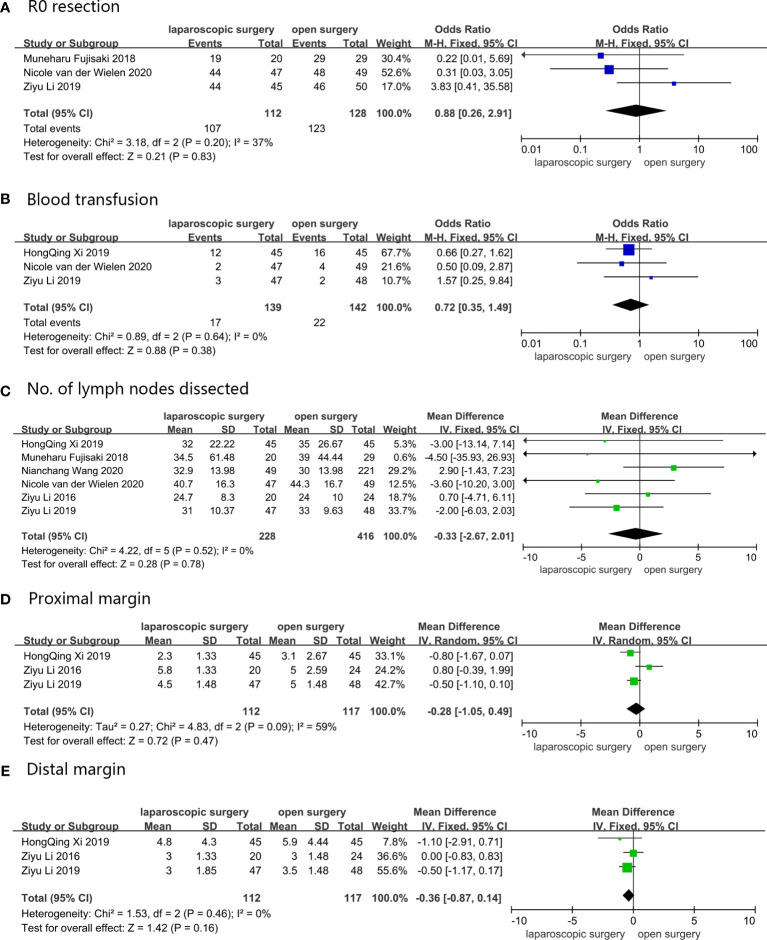
Intraoperative conditions: **(A)** R0 resection; **(B)** Blood transfusion; **(C)** No. of lymph nodes dissected; **(D)** Proximal margin; **(E)** Distal margin.

Three studies reported R0 resection, which was defined as a microscopic margin-negative resection with tumor-free margin greater than 1 mm (27, 30, 31). Results obtained from the pooled analysis showed that 107 (95.5%) out of the 112 AGC patients who underwent LG received R0 resection, while 123 (96.1%) out of 128 AGC patients who received OG received R0 resection, with OR = 0.88, 95%CI: 0.26–2.91, p = 0.83, and a moderate heterogeneity (I^2^ = 37%). Blood transfusion was recorded in three studies (27, 31, 32), with the pooled results indicating that 17 (12.2%) out of 139 AGC patients who were assigned to LG required blood transfusion, and 22 (15.5%) out of 142 AGC patients who underwent OG received blood transfusion (OR = 0.72, 95%CI 0.35–1.49, p = 0.64, and I^2^ = 0%). All six studies described intraoperative blood loss and the number of dissected lymph nodes (27–32), with the weighted mean differences for intraoperative blood loss and the number of dissected lymph nodes of LG versus OG being -8.76 ml (CI: -28.17–10.65, p = 0.38, and I^2^ = 36%) and -0.33 (CI: -2.76–2.01, p = 0.78, and I^2^ = 36%), respectively. Proximal and distal margins, which were defined as distance from the proximal or distal resection margin to the edge of the tumor area, were reported in three studies (27, 28, 31), and the weighted mean differences were -0.28 mm (95%CI: -1.05–0.49, p = 0.47, and I^2^ = 59%) and -0.36 mm (95%CI: -0.87–0.14, p = 0.16, and I^2^ = 0%), respectively. The pooled results showed no change for all intraoperative conditions when sensitivity analyses were performed, and the conversion between random-effects and fixed-effects model did not influence the pooled results using RevMan5.4.1 software. These results are shown in **Figure 2**.

### Postoperative Short-Term Clinical Outcomes

**Figure 3:**
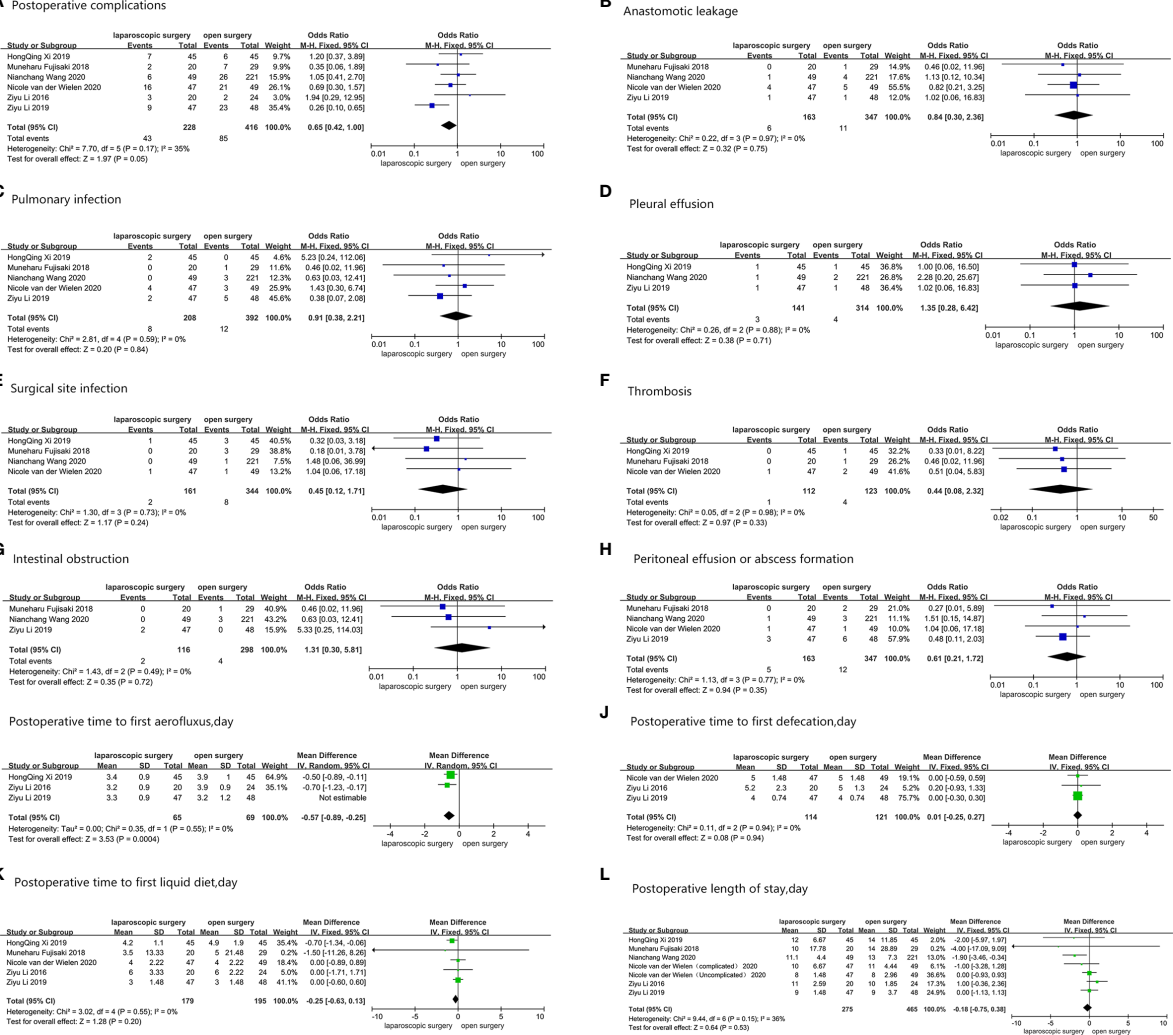
Postoperative short-term clinical outcomes: **(A)** Postoperative complications; **(B)** Anastomotic leakage; **(C)** Pulmonary infection; **(D)** Pleural effusion; **(E)** Surgical site infection; **(F)** Thrombosis; **(G)** Intestinal obstruction; **(H)** Peritoneal effusion or abscess formation; **(I)** Postoperative time to first aerofluxus, day; **(J)** Postoperative time to first defecation, day; **(K)** Postoperative time to first liquid diet, day; **(L)** Postoperative length of stay, day.

All six studies provided the number of patients who developed postoperative complications (27–32). Pooled results displayed that 43 (18.9%) out of 228 AGC patients experienced postoperative complications after LG, while 85 (20.4%) out of 416 patients who underwent OG experienced postoperative complications, with OR = 0.65 (95%CI: 0.42–1.00, p = 0.05, and I^2^ = 35%). **Figure 3** shows results obtained after comparing both groups with regard to all common complications, including anastomotic leakage (27, 29, 30, 32), pulmonary infection (27, 29–32), pleural effusion (27, 29, 31), surgical site infection (29–32), thrombosis (30–32), intestinal obstruction (27, 29, 30), and peritoneal effusion or abscess formation (27, 29–32). Three studies reported the postoperative time to first aerofluxus (27, 28, 31), and the mean difference between the LG group and the OG group was -0.57 day (95%CI -0.89–0.25, p = 0.0004, and I^2^ = 0%) (27, 28, 31). Three studies described the postoperative time to first defecation (27, 28, 32), and the mean difference was 0.01 day (95%CI -0.25–0.27, p = 0.94, and I^2^ = 0%). Five studies reported the postoperative time to first liquid diet (27, 28, 30–32), and the mean difference between the two groups was -0.25 day (95%CI -0.63–0.13, p = 0.2, and I^2^ = 0%). The postoperative length of stay was reported in all six studies, and the mean difference was -0.18 day (95%CI -0.75–0.38, p = 0.53, and I^2^ = 36%). The pooled results showed no change when sensitivity analyses were performed, with exception of postoperative complications, distal margin, and postoperative time to first aerofluxus. Moreover, the conversion between random-effects and fixed-effects model did not influence all the pooled results using RevMan5.4.1 software. For postoperative complications, no significant bias was found in the study by Li et al. (27); thus, we retained the study during pooled analysis of postoperative complications. However, for postoperative time to first aerofluxus, the authors reported a more conservative and cautious diet management (27), which may have influenced the pooled result. Thus, the study was removed from the pooled analysis. **Figure 3** shows all pooled results of postoperative short-term clinical outcomes.

### Postoperative Survival Outcomes

**Figure 4:**
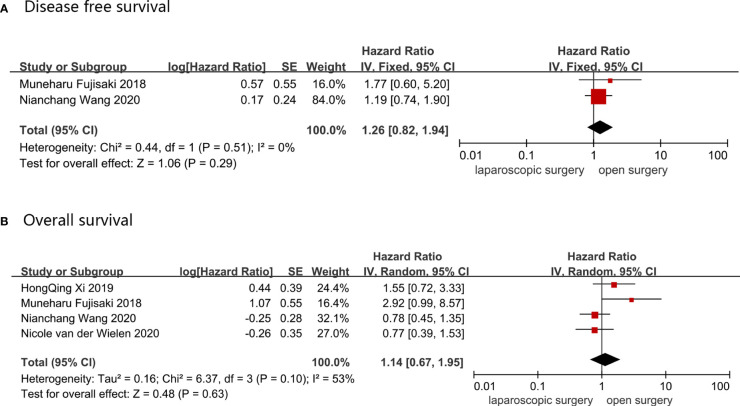
Postoperative survival outcomes: **(A)** Disease free survival; **(B)** Overall survival.

OS was reported in four studies, which comprised a total of 505 AGC patients, of whom 161 underwent LG and 344 received OG. A random-effect model was used to analyze the OS data because the heterogeneity was 53%, indicating a high heterogeneity, and the HR value for OS was 1.14 (LG *vs.* OG, 95%CI: 0.67–1.95 and p = 0.63). DFS was reported in two studies that involved 319 AGC patients, of which 69 underwent LG and 255 received OG. Data was synthesized using a fixed-effect model, and DFS HR (95%CI) value was 1.26 (0.82–1.94), with p = 0.29 and I^2^ = 0%. There was no change in the pooled results when sensitivity analyses were performed, and the conversion between random-effects and fixed-effects model did not influence the pooled results using RevMan5.4.1 software. These results are shown in **Figure 4**.

## Discussion

There is an urgent need to evaluate the safety and efficacy of performing LG in AGC patients following NAC due to the expanding use. For AGC patients who received NAC, it is inappropriate to directly use results reported in previous systematic review and meta-analysis studies, which compared safety and efficacy of LG versus OG in AGC patients who did not receive NAC (38–41) because they ignored the impact of NAC on the surgical procedure (42). The studies provide unreliable evidences that may extremely scale up or scale down safety and efficacy differences between LG and OG. Therefore, it is vital to summarize current evidences in order to guide the choice of surgical procedure for AGC patients following NAC. In this meta-analysis, we performed a pooled analysis, which compared the efficacy and safety of LG versus OG for AGC patients who received NAC before radical surgery.

For intraoperative conditions, both LG and OG reached high R0 resection (more than 95%), and there were no significant differences between the LG group and the OG group. An important and meaningful point was to determine whether LG showed sufficiently high R0 resection rate that was comparable to OG, because fibrosis and edema of cancer foci after NAC had an impact on the surgical safety and efficacy (42). The American Joint Committee on Cancer (AJCC) recommended that at least 15 lymph nodes should be examined for GC patients to ensure accurate and robust N staging (43). Recent studies have revealed that AGC patients with dissection of more than 25 lymph nodes had longer survival time (44), and dissection of more than 29 lymph nodes enabled maximization of survival benefit for AGC patients (45). The results of this meta-analysis have shown that the mean number of lymph nodes resected in the LG group exceeded 29 lymph nodes, and there was no significant difference in the number of dissected lymph nodes between LG and OG for AGC patients with NAC. Intraoperative blood loss and intraoperative blood transfusion results were not consistent with findings reported in previous studies (14, 16, 46), and they showed no significant differences between the LG group and the OG group. One possibility for these discordant results was that NAC caused lesional tissue edema and fibrosis, which added to the difficulty of hemostasis performed by laparoscopic surgery (17–26). Moreover, there were no significant differences in the proximal and distal margin between LG and OG patients, which was perhaps because proximal and distal margin mainly depended on the technical proficiency of the surgeon and biological characteristics of tumor.

With regard to postoperative short-term clinical outcomes, the LG group showed much less postoperative complications than the OG group for AGC patients with NAC, which was consistent with the findings reported in the included RCT study (27). This can be attributed to the intrinsic advantages of laparoscopic surgery in terms of clear surgical view and mild surgical manipulation, which makes manipulating organ, and dissociating nerves and blood vessels easier. Another probable reason is that the application of sophisticated equipment during laparoscopic surgery such as the LigaSure vessel sealing system (LVSS) and the harmonic scalpel decreases the surgery damage inflicted to the normal tissues, thereby reducing postoperative complications (47). Furthermore, there were no significant differences in a variety of factors that could influence the postoperative short-term clinical outcomes including sex, body mass index (BMI), American Society of Anaesthesiology (ASA) scores, objective response rate (ORR), clinical TNM stage, ypT3 or T4 stage, ypN2 or N3 stage, resection range, reconstruction approach, and tumor longitudinal between the LG group and OG group (48, 49). Although a previous study demonstrated that LG was capable of reducing the incidence of pulmonary and cardiovascular adverse events (50), results obtained in this study showed that the incidence of pulmonary infection, pleural effusion, and thrombosis were strikingly low (≤5%), and there were no significant differences between the LG group and the OG group for AGC patients with NAC. We hypothesize that this difference could be attributed to improvement of postoperative nursing, where medical staff encouraged and instructed patients to employ integrated control measures, including effective cough and expectoration, inhalation of sputum *via* aerosolized droplets, and early out-of-bed mobilizations. Previous studies have reported that anastomotic fistula is predominantly associated with surgical equipment and proficiency of the surgeon (51–53), and different surgical approaches have less impact on the risk of developing anastomotic fistula. In this meta-analysis, the incidence of anastomotic fistula was low in both the LG group and the OG group (≤5%), and it was comparable between the two groups. A previous study reported a significantly decreased incidence of postoperative infections due to the use of perioperative antibiotic prophylaxis (54). In this study, results showed that no significant differences were found in the occurrence of peritoneal effusion or abscess formation and surgical site infection between the LG group and the OG group. Surgical manipulations will stimulate the gastrointestinal tract nerves, reflexively resulting in inhibition of gastrointestinal peristalsis. Therefore, the extent of inhibition was greatly associated with proficiency of gastrointestinal surgeons rather than surgical ways (53), and our pooled result of the incidence of intestinal obstruction was comparable between the LG group and the OG group (p≤0.05). Previous clinical trials consistently reported that AGC patients with LG who had not received NAC had a quicker recovery of bowel function, including faster postoperative time to first aerofluxus, postoperative time to first defecation, postoperative time to first liquid diet, and a shorter hospital stay (13, 14, 55, 56). Results revealed that, with exception of postoperative time to first aerofluxus, there were no significant differences in postoperative time to first defecation, postoperative time to first liquid diet, and hospitalization time between the LG group and the OG group. The lack of statistical differences may be because chemotherapeutic drugs have effects on recovery of bowel function and the unblended study design introduced substantial information bias. Moreover, the lack of high-quality evidences supporting the application of LG in AGC patients with NAC may make surgeons to be more conservative and cautious in diet management and hospital discharge criteria of postoperative patients, which partially explains why our results were inconsistent with results reported in previous studies.

With regard to postoperative survival outcomes, there were no significant difference in DFS and OS between the LG group and the OG group for AGC patients with NAC. A previous study indicated that when surgical margins fulfilled R0 resection criteria and the number of removed lymph nodes was sufficient, the intrinsic biological characteristic of gastric cancer greatly determined the survival time (57).

## Conclusion

Overall, LG was an effective and safe treatment approach for AGC patients with NAC, and LG and OG were comparable in intraoperative conditions, postoperative short-term clinical outcomes, and postoperative survival outcomes. Moreover, LG exhibited lower postoperative complication rate compared to OG. These results suggest that surgeons should perform LG for AGC patients who receive NAC.

## Limitations

Some important limitations were presented in this meta-analysis. On the one hand, the study only contained six original studies involving 644 patients, which may have led to false negative results. On the other hand, some uncontrollable factor differences existed among studies. For example, both NAC regimens and the surgeon proficiency for LC and OG varied among the included studies, which may have introduced substantial bias. In addition, the younger patients in the LG group may have had better health than patients in the OG group. Therefore, high-quality, multicenter, and large sample RCT studies should be urgently performed to confirm our findings.

## Data Availability Statement

The original contributions presented in the study are included in the article/supplementary material. Further inquiries can be directed to the corresponding author.

## Author Contributions

X-LL and X-WL contributed equally to the paper. X-LL and H-YP conceived and designed the study. All authors were responsible for data collection, analysis, interpretation, and generation of figures, and X-LL and X-WL were involved in writing of the paper. ZW-H took part in paper checking and modification. J-KH gave final approval of the manuscript. All authors contributed to the article and approved the submitted version.

## Funding

1. This work was supported by Sichuan Science and Technology Program (No.20YYJC3357, No.2019YFS0255, No.2021YJ0475).
2. Project funded by China Postdoctoral Science Foundation (2019M653418, 2020T130449)

## Conflict of Interest

The authors declare that the research was conducted in the absence of any commercial or financial relationships that could be construed as a potential conflict of interest.

## Publisher’s Note

All claims expressed in this article are solely those of the authors and do not necessarily represent those of their affiliated organizations, or those of the publisher, the editors and the reviewers. Any product that may be evaluated in this article, or claim that may be made by its manufacturer, is not guaranteed or endorsed by the publisher.

## Abbreviations

AGC, Advanced Gastric Cancer; NAC, Neoadjuvant Chemotherapy; LG, Laparoscopic Gastrectomy; OG, Open Gastrectomy; DFS, Disease-free Survival; OS, Overall Survival; JCS, Jadad Composite Scale; NOS, The Newcastle Ottawa Quality Assessment Scale.
